# Radial basis function for fast voltage stability assessment using Phasor Measurement Units

**DOI:** 10.1016/j.heliyon.2019.e02704

**Published:** 2019-11-14

**Authors:** Jorge W. Gonzalez, Idi A. Isaac, Gabriel J. Lopez, Hugo A. Cardona, Gabriel J. Salazar, John M. Rincon

**Affiliations:** Universidad Pontificia Bolivariana – Medellin, Antioquia, Colombia

**Keywords:** Electrical engineering, Electrical system planning, Power engineering, Electric power transmission, Power system operation, Power system planning, Power system stability, Phasor Measurement Units, Voltage measurement, Radial basis function networks, Machine learning

## Abstract

A simple method, based on Machine Learning Radial Basis Functions, RBF, is developed for estimating voltage stability margins in power systems. A reduced set of magnitude and angles of bus voltage phasors is used as input. Observability optimization technique for locating Phasor Measurement Units, PMUs, is applied. A RBF is designed and used for fast calculation of voltage stability margins for online applications with PMUs. The method allows estimating active local and global power margins in normal operation and under contingencies. Optimized placement of PMUs leads to a minimum number of these devices to estimate the margins, but is shown that it is not a matter of PMUs quantity but of PMUs location for decreasing training time or having success in estimation convergence. Compared with previous work, the most significant enhancement is that our RBF learns from PMU data. To test the proposed method, validations in the IEEE 14-bus system and in a real electrical network are done.

## 1 Introduction

When dealing with the stability of a power system, reference is made to the ability of preserving electromechanical conditions within appropriate bounds, even after a disturbance has occurred. Currently, electric power systems operate under an increasing energy demand, with electrical power transmission infrastructure growing in a lower rate due to environmental concerns, in general. Then, the ability of power systems to maintain voltage stability is gradually becoming a major issue. Faced with this increasing demand, utilities have to redouble efforts to keep voltage stability and perform studies around feasible disturbances that may lead to potential collapses. From results of simulations, preventive and corrective measures could be undertaken for maintaining normal operation. In this way, recent studies on the operation and planning of electric power systems show that voltage stability is one of the main challenges that must be faced when guaranteeing the security of a system.

For years, voltage stability has been studied by system operators due to the consequences of a series of events in different countries [1, 2, 3, 4]. These events are still a motivation for researchers to develop methods and online applications to aid in fast and accurate voltage collapse prediction.

The methods of solving this problem can be divided into two main categories. As a first option, it consists of carrying out a preventive analysis to know the limits in which the system can be maintained in a normal and steady operation. As a second option, it is necessary to identify the corrective controls that can be carried out in order to return back the system to its normal operation, after a disturbance. Another proposed solution that is offered to voltage stability assessment is to guarantee the generation of reactive power as a margin of reserve in those points of the weakest network, where it is estimated that a variation in the reactive load can cause voltage instabilities.

Several studies allow to measure and predict the conditions of the system with respect to the stability of voltage. This is done in order to prevent collapses in power systems due to lack of stability after a disturbance takes place. To mitigate the undesirable events of voltage instability, direct measures or analyses are applied on these estimates.

In [5], an online voltage security assessment method based on wide-area measurements and decision-tree algorithm was proposed for fast evaluation. For near-real time voltage stability analysis [6], proposed a voltage stability and conditioning monitor, model-free in real-time, based on Phasor Measurement Units, PMU. In [7, 8], authors provided Thevenin equivalent methods for fast voltage stability assessment. Reference [9] applied fuzzy inference to estimate loading margins for voltage stability in real-time operation. In [10], a method for selection of the most effective controls to prevent voltage instability was proposed. A local index for online estimation of closeness to loadability limits using two consecutive measurements is presented in [11]. Authors in [12] proposed a fast method to calculate voltage stability security margins based on nonlinear programming techniques. In the field of voltage security control [13], suggested a method for applying preventive controls against voltage instability in the presence of multiple critical contingencies. In [14] a modification of L-index is proposed to include generators for accurate online voltage analysis.

Diversity in methods listed, confirm that the subject of online voltage stability assessment is pertinent at present. In this work, we will propose a novel method.

Regarding voltage stability indices for online applications [15], determines voltage stability limits after network reduction to two nodes. Reference [16] presented an index for administrative control areas to monitor quasi-static voltage instability at the interconnection level. In [17], it is presented an index of zones to monitor voltages in real time. Paper [18] proposed a voltage security index with PMUs. Reference [19] offers an approach on wide area voltage stability control by remedial actions. As a complement, global and zone indices will be defined and implemented in this work.

To realize fast voltage stability prediction, machine learning has techniques widely adopted as alternative approaches in recent years. Artificial Neural Networks, ANN, have often been used for voltage stability strategies. In particular, Multi Layer Perceptron, MLP, ANN counts on many applications. Radial Basis Functions are conceived as a special kind of ANN that belongs to Machine Learning strategies. In some papers, RBF is termed as the better choice of ANN, and based on experiences, it has been concluded that this kind of network would be preferred to MLPs due to fast training time, simple structure and accuracy on estimation [20, 21, 22, 23, 24, 25]. RBF proposals for voltage stability assessment have been reported in the literature. In [26], it is proposed a method for voltage stability evaluation, where RBFs respond better than MLPs. Reference [27] recollected a current state of ANN applications in power system and reported a wide use of RBFs for static and transient security assessment. In [28], it is presented an approach with RBFs to rank expected contingencies that may cause steady state voltage violations and highlight the training efficience of RBFs. Paper [29] suggests a scheme for online voltage stability monitoring for multiple contingencies with RBF. In [30] a control scheme with RBFs is presented to provide voltage compensation in microgrids. In this work, we will then propose a method using RBFs for fast voltage stability estimation including PMUs.

In today's traditional supervisory systems, it is common to count on different stations measurements lacking of a common timestamp. This situation is being resolved nowadays integrating new monitoring equipment such as PMUs to observe wide areas, ensuring data synchronization [31, 32]. Some developments based on PMUs have used bus voltage magnitude, phase angle and current through a circuit to calculate indices and find loading margins or distance to voltage collapse point [33, 34]. The methods developed showed that, alongside voltage magnitudes, surprisingly the inclusion of phase angles also helped in voltage stability margins forecast. We will then add voltage angles in our method.

There are works on voltage stability indices with PMU measurements, some intended for real-time applications, [35, 36, 37]. Some indexes are not suitable to any power system topology and are only accurate for radial networks [38, 39] and exhibit large deviations or require complex computational processes when the system is meshed. Projecting renewable sources integration, the expansion plans in the Colombian power transmission system have allowed interconnecting areas that were radial in the past. The proposed method in this paper will take into account this new behavior of power systems, but also considering radial topology in some areas.

For cost reasons and large data management, it is important to optimize the number of PMUs installed. Methods taking advantage of a least quantity of PMUs are always welcome [40, 41, 42]. However, it will be found in this work, that the minimum quantity of PMUs is not a warrant of voltage stability assessment success or training time decrease, but of PMUs location. This was not sensed in cited works.

The main contributions of this work are as follows:

• This work is an attempt to propose a method based on RBFs for a fast estimation of voltage stability margins in real-time operation including bus voltage magnitudes and phase angles, with the fewest quantity of PMUs installed in strategic locations. The system framework is significantly simple to implement.
• Compared with previous work [26], the most significant enhancement is that our RBF learns from PMU data, which is promissory for multiplicity of wide area applications. Reference [26] does not count on synchronized angles from PMUs and, of course, no PMU optimized allocation was endeavored.
• It is proposed a global voltage stability margin to be estimated for the whole system and for each load bus with complete network and N-1 contingency criteria.
• It is found that the minimum quantity of PMUs is not a warrant of voltage stability assessment success or training time decrease, but of PMUs specific location. This was not sensed in previous works for voltage stability concerns.
• The proposed system is assessed in the benchmark IEEE 14-bus system and in a real network. The simulation results demonstrate accurate evaluation with significantly reduced system response time.

The rest of the paper is organized as follows. After the introduction, the model formulation is given. The proposed methodology, simulations, results from case studies and the discussions are presented next. The paper ends with the conclusions and references.

## 2 Model

### 2.1 Optimal PMU placement to ensure full observability

Based on the methodology used in [40, 41, 42], an optimization function can be defined and solved with binary integer programming to find a minimal set of PMUs, such that a bus is observed at least once.

The optimal placement of PMUs for an N bus system is then formulated (1)

(1) $$Min\sum_{k=1}^{N}W_{k}X_{k}$$

where

• *N* is the total number of nodes in the grid
• *W*_*k*_*=1* (function cost to install a PMU).

Subjected to the restriction in (2)

(2) AX≥b

*A* is the binary connectivity matrix and *b* a binary vector with size Nx1, where 0 represents ZIB's (zero injection buses) substations and 1 other cases.

*X* is a binary variable vector whose entries are 1 if a PMU is needed at bus *k* and 0 otherwise. That is why the size of this vector is Nx1. See (3).

(3) $$X={\left[X_{1}X_{2}...X_{N}\right]}^{T}$$

To solve the problem, binary integer programming function is used. Matlab [43] allows solving this to find X vector.

**Fig. 1:**
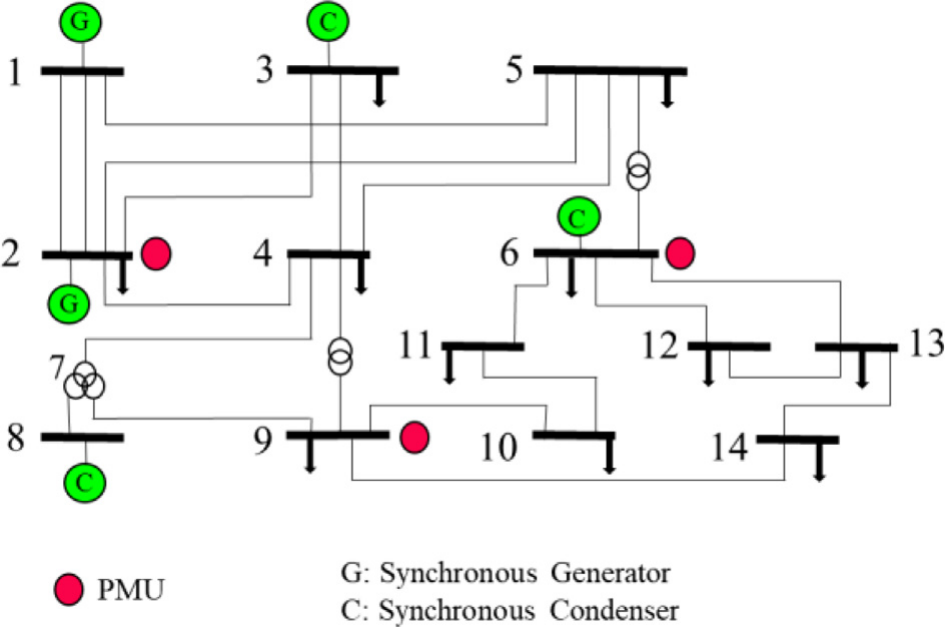
IEEE 14 bus bar system with PMU optimized placement.

As an example of the optimized observability algorithm application, IEEE 14 bus network is used. Only 3 PMUs located at buses 2, 6 and 9 are required to make observable the network. Bus 7 was not considered as it is an auxiliary node, see Fig. 1.

### 2.2 Voltage stability margins calculation

One factor for the security of a power system is to predict the remaining reserves. This can be done by using tools to prevent and analyze security and system status [44, 45, 46, 47].

**Fig. 2:**
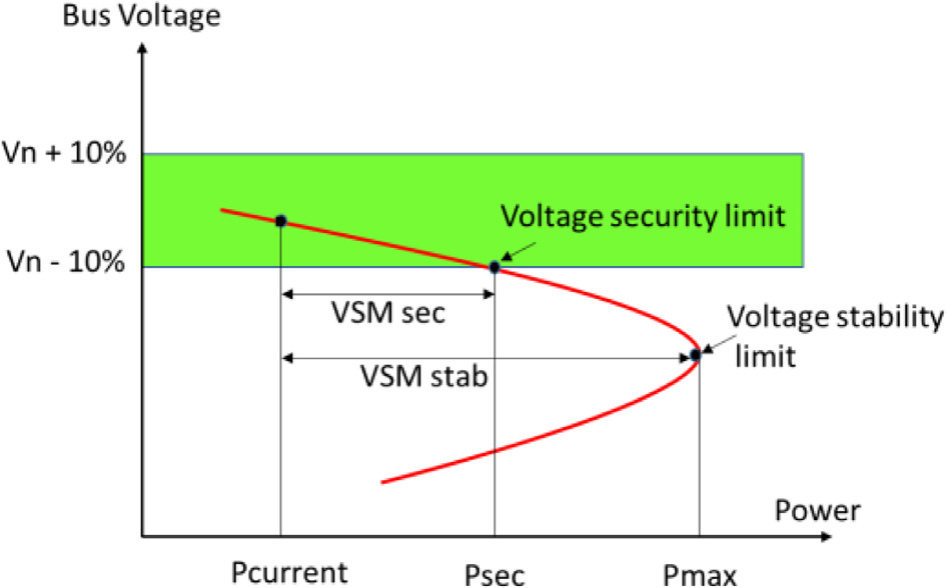
Definitions for long-term voltage stability assessment.

For long-term voltage stability [48, 49], the Continuation Power Flow, CPF, is performed with a generation and demand increase distributed in all loads, not focusing on specific areas. However, this can be modified in the method according to desired load increase behavior or direction. From this stage, global voltage collapse conditions are determined and PV curves method is used. With PV curves, a voltage security criterion is established for all buses defining an allowable voltage range, e.g., ±10%, for pre and post-contingencies. This can be observed in Fig. 2.

Two Voltage Stability Margins, VSM, are defined in this work for a load bus *i* according to Fig. 2. The first, which is a situation compromising the loss of attraction of the system, corresponds to the arithmetic difference between voltage stability limit, defined by Pmax, and the current load demand, see (4). The second one corresponds to a voltage stability limit for secure operation. This is for a point in which a bus voltage is located in the lower bound allowed by the power system operator, e.g., nominal voltage, Vn, minus 10%. See (5).

(4) $$VSM_{stab\_i}=P_{\max}-P_{current}$$

(5) $$VSM_{\sec\_i}=P_{\sec}-P_{current}$$

Two global margins are defined, a global voltage stability and a global secure margin. See (6) and (7). These limits yield the total margin of the power system before any bus reaches a stability margin.

(6) $$VSM_{stab\_global}=\sum \left(VSM_{stab\_i}\right)$$

(7) $$VSM_{\sec\_global}=\sum \left(VSM_{\sec\_i}\right)$$

### 2.3 Radial Basis Functions

**Fig. 3:**
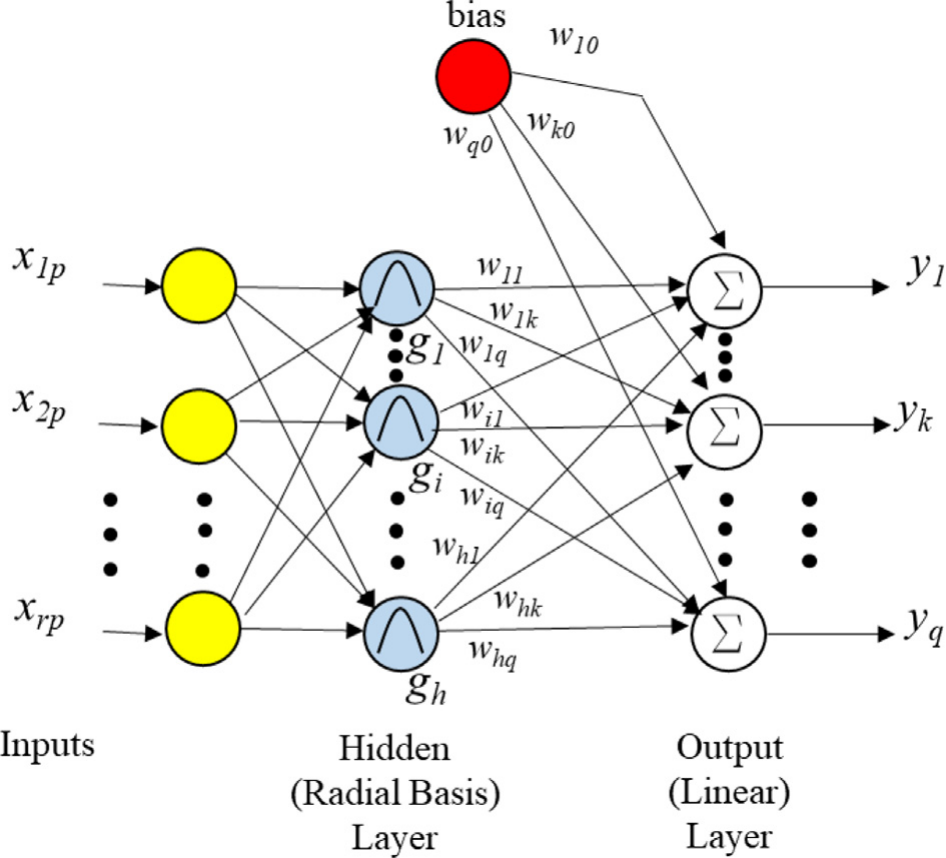
Architecture of Radial basis function, RBF.

ANNs can detect complex nonlinear relationships. This application will be exploited to estimate load margins. As indicated, RBFs, are part of Machine Learning methods and are a kind of ANN that has become increasingly used because of its simplicity in structure and training efficiency. The architecture is shown in Fig. 3.

RBFs count on the advantage of fast learning and ability to detect outliers during estimation. The attractive feature of RBFs is the linear dependence in the parameters. This simplifies in great manner the design and analysis. It also has an advantage of an easy and effective learning algorithm compared to MLP ANNs [26]. RBFs could demand more neurons, but it is typical that RBFs be designed in much less time than the classical standard feed-forward backpropagation networks and MLPs. RBFs are also trained in less time, demanding low computational resources. Obviously, they work better when many training vectors are available [50].

According to Fig. 3, RBFs consist of two layers: a hidden radial basis layer, and an output linear layer. The hidden layer involves neurons with non-linear functions named basis functions, in which the arguments represent the Euclidean distance between applied inputs patterns and the centre of the basis function. The Gaussian exponential function is normally used for the basis function as in (8).

(8) $$g_{i}\left(x\right)=\exp\left(-\frac{\|x-\mu_{i}{\|}^{2}}{2\sigma_{i}^{2}}\right)$$

Where *x* is the input vector and $\mu_{i}$ is the weight vector associated with the hidden unit *i* (which is the center of the Gaussian exponential function). The expresion $x-\mu_{i}$ is the Euclidean distance between the input vector x and the centroid subvector $\mu_{i}$; *σi* is the spread parameter of the radial basis function. The vector *x* is composed of *r* rows or inputs. Each neuron catches the Euclidean distance between the input array and neuron's centroid and transports the resulting scalar through the nonlinear function g(x). The name given as “Radial Basis” lies in the fact that the inputs with the same distance to the centroids obtain the same output result. The spread allows the sensitivity of the Gaussian function to be adjusted. In Matlab [51], the RBF takes the spread as a bias. The value of the *kth* output node *yk* is given in (9).

(9) $$y_{k}\left(x\right)=\;\sum_{i=1}^{h}w_{ik}\;g_{i}\left(x\right)+w_{k0}$$

**Fig. 4:**
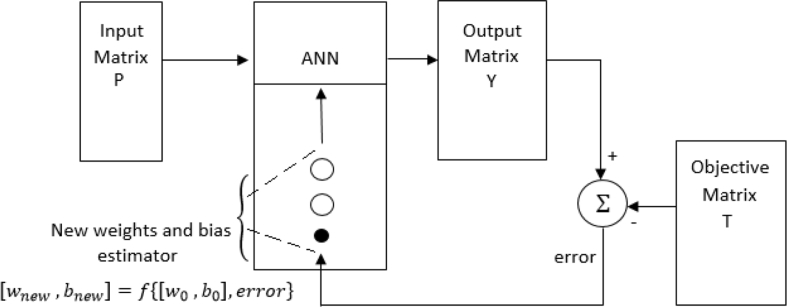
System with ANN and training process with matrices P and T.

Where $w_{ik}$ is the weight connecting *ith* neuron in the hidden layer to the *kth* output neuron. The bias $w_{k0}$ adjusts the sensitivity of the radial basis neuron. For RBF construction and training we used the Matlab Neural Network Toolbox [51]. It is possible to accomplish a supervised learning as shown in Fig. 4. Input data is the matrix P; results from the RBF are recollected in output matrix Y and target matrix is T. The matrix P is treated as a set of input vectors ‘*x’.* Matlab returns a network with weights and biases so that P inputs produces T objective. One neuron is created at a time, but neurons are provided until the Mean Square Error, MSE, falls below a target error or a maximum number of neurons is reached. In next sections, detail explanation of matrixes P and T construction will be presented.

## 3 Methodology

**Fig. 5:**
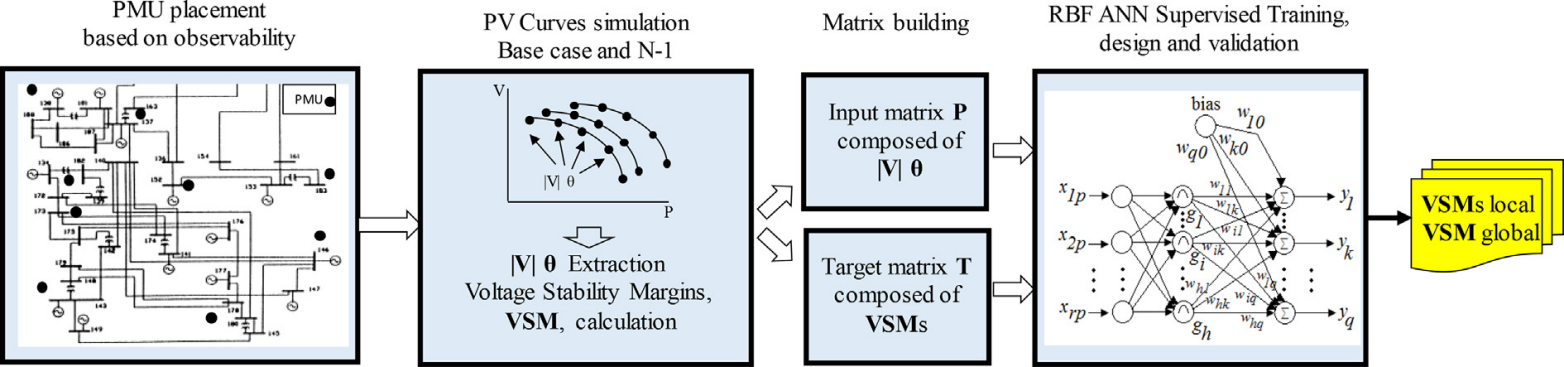
Proposed Method for Voltage Stability training with RBF and a reduced set of Voltage Phasors.

The proposed method should perform real-time analysis of voltage stability state by monitoring loadability margins. The algorithm can be visualized in Fig. 5. Input data is obtained by locating PMUs by the optimization procedure described in Section 2.1. Then, steady state voltage stability analysis yields PV curves of load nodes.

**Fig. 6:**
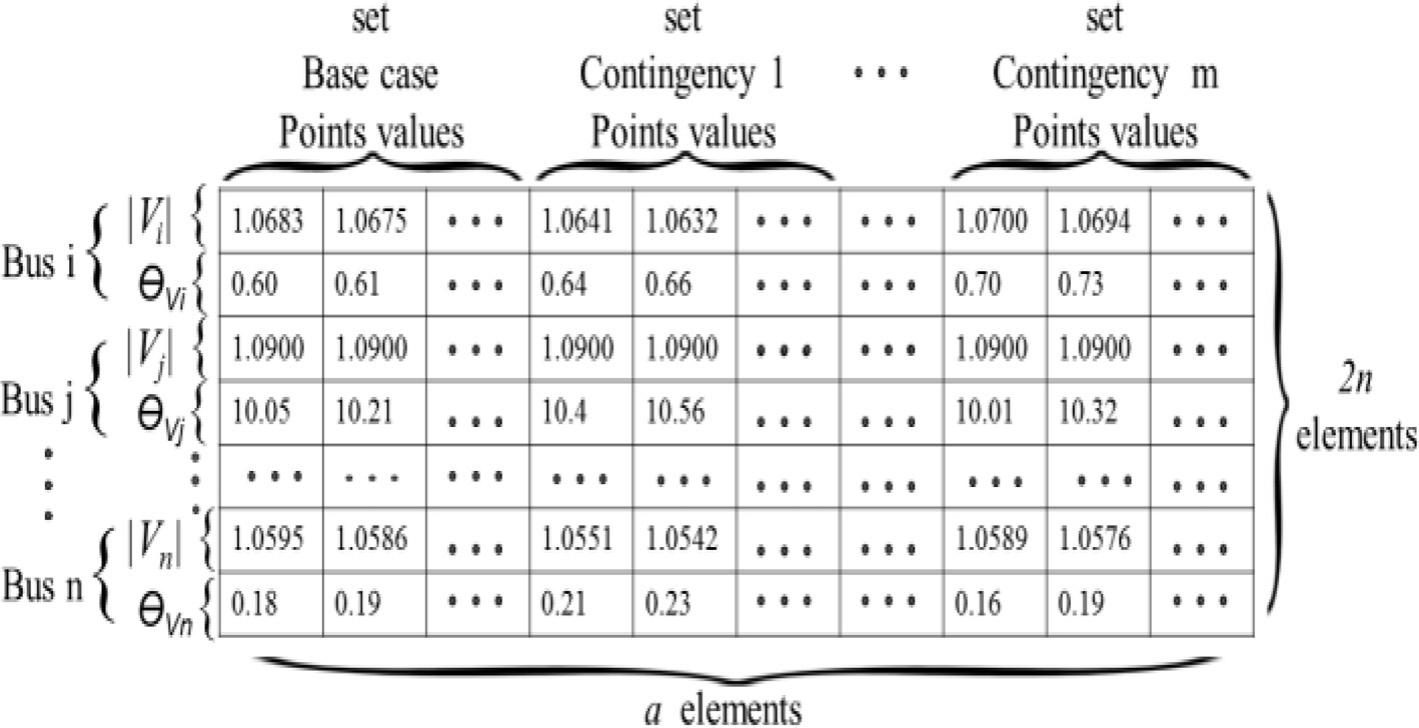
Input matrix P for the RBF.

Results are stored in matrix P, which behaves as the knowledge base of input features for the RBF. P lumps together the operating points composed by bus voltage magnitudes and phase angles at each operational point considered. P is shown in Fig. 6 for an arbitrary case. Each set belongs to a particular scenario, as the base case or cases with diverse contingencies. Each set is composed of multiple points taken along a PV curve as in Fig. 5. The points of a set should be separated by a common amount of megawatts along the PV curve. P will then have *r* = *2n* rows, being *n* the total number of buses, and *a* columns. The quantity *a* can total a large number of values depending on the load step between the points of each scenario set, i.e., the base case and contingencies. Each column is correlated to a point of a PV curve. The final number of rows of P will depend on the quantity of PMUs. Each row has all the PV curves for each bus and case considered. In this work, cases were performed locating PMUs in different quantity of buses, including the case of optimized placement. P is treated as a set of *a* input vectors ‘*x’* for the neural network as in Fig. 3.

**Fig. 7:**
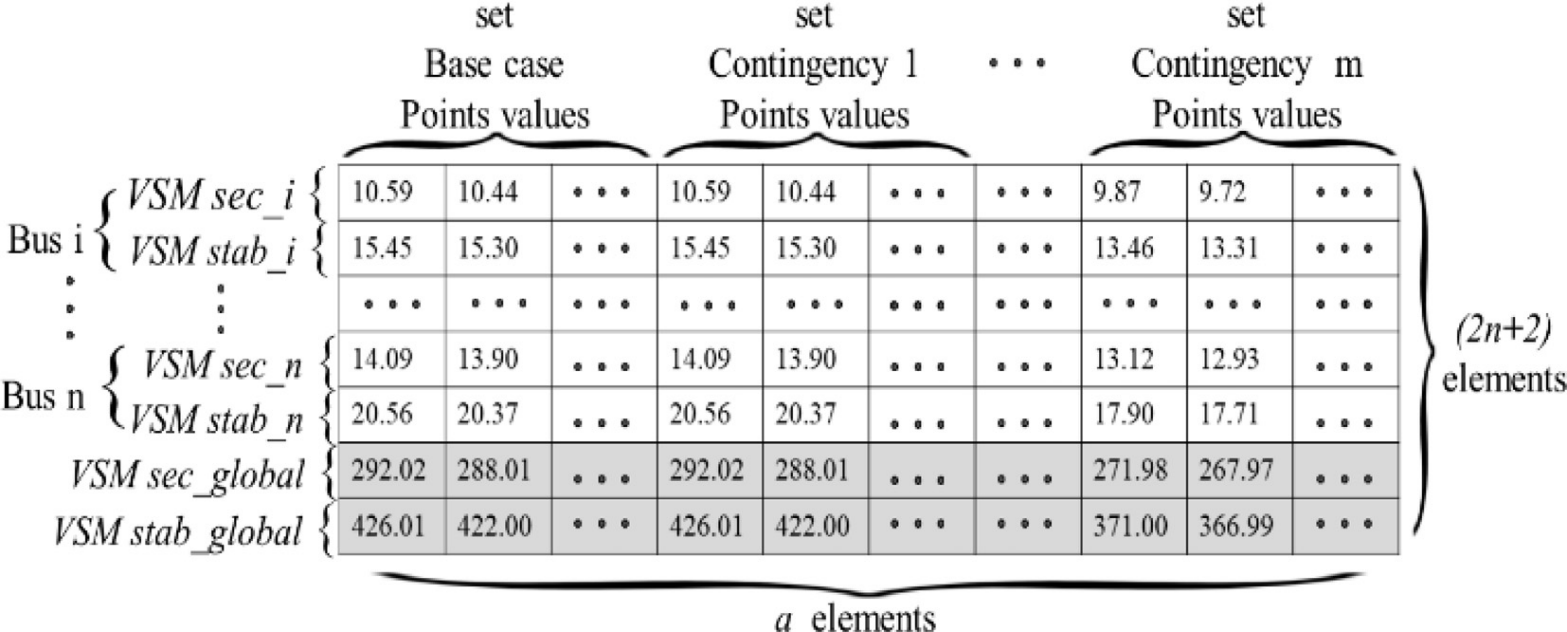
Target matrix T for the ANN.

With PV analysis, target expected VSMs are then calculated with (4) to (7) and stored in target matrix T. This matrix, shown in Fig. 7, has the objectives from the RBF after training. The order of this matrix is *(2n + 2)*x*a*. This is because it is composed by the secure and stability margins for each bus as defined by (4) and (5), and complimented in the lowest two rows by the two global margins of (6) and (7). The number of columns of T equals the columns of P, because scenario sets and points used are the same. Matrix T is the comparator for the output matrix Y, as in Fig. 4. During training, an error between simulated and expected results is obtained. This error is used as a further input to iteratively adapt weights *W* and bias *b,* and set up a system capable of producing the expected results. See (10).

(10) $$\left[W_{new},b_{new}\;\right]=f\left\{\left[W_{0},b_{0}\;\right],\;error\right\}$$

Training finishes when output and targets values are reasonably comparable according to MSE. Finally, a validation is executed, verifying that a data sample initially extracted from matrix P and not used in the training process, can produce the expected results.

When testing in machine learning, there are two concerns to be balanced: with less data for the training, the parameter estimations have greater variance. With less data for testing, the performance statistic will have greater variance. One should be concerned with dividing data such that neither variance is too high, which is more to do with the strategic or absolute number of instances in each category rather than percentages. There could be an intermediate balance, but as a number it is not rigorously established. Some authors establish that these ratios are subjective and then propose researches. It is sometimes pointed out that the fraction of patterns reserved for the validation set should be inversely proportional to the square root of the number of free adjustable parameters. In their conclusions, they propose formulas based on the number of families of recognizers and the complexity of those families. What they mean by complexity is that each family of recognizer is characterized by its complexity, which may or may not be related to the dimension, the description length, the number of adjustable parameters, or other measures of complexity.

Accusing to the concept of families, in this work each designed RBF was trained considering operating points for a base case and contingencies. Each of these cases produce a different PV curve or family. The total of points gathered would include certain number of PV curves of families for the base case and contingencies. The points used for validation, comprising voltage magnitude and angles in busbars with PMUs, can be strategically selected with criteria involving intentions for evaluating aspects devoted to what is explored. For example, that each point represents a different topological condition or family of the system, trying in addition that points could reflect diverse qualities for the stability of the system, conditions in the medium range of stability and conditions in ranges close to the loss of stability where the system is mathematically complicated. In this way, each curve produces a common behavior of the system or interrelation between points. That is why the points were selected with the philosophy described before; belonging to different PV curves. This strategy can decrease the number of points for testing the ANN and avoid choosing big groups of points that may be concentrated in a reduced number of topologies, losing diversity. Now, the RBF may be ready for real time supervision.

To show the convenience of the methodology, in which the emphasis is to have a minimum set of PMUs strategically placed, VSMs are initially estimated considering PMUs at all buses of the grid. The next stage in ANN training will consist in a sensitivity around the minimum number of PMUs required for a margin estimation. On first, a random placement of few PMUs is done. Then, full observability of the system is applied to determine the minimum amount of PMUs to observe the system. Comparisons between the two cases are done. Different RBFs are trained verifying its behavior according to the MSE. The studies include, number of PMUs, number of neurons and MSE. Results will expose how the location of PMUs could be strategic for the performance.

## 4 Examples

The IEEE 14-bus system is used to explain the proposed methodology. The first part of the process consists on optimally locating the PMUs based on observability theory. In Section 2 it was found a solution with PMUs placed in buses 2, 6 and 9. However, to analyze the effects of placing PMUs in an optimized manner, for the voltage stability estimation of our method, comparisons of installing PMUs in all, four and two buses are done. The next step is PV curves study. The network is modified in generation to establish a large number of operating points without voltage problems. See Table 1.

**Table 1:** Modified generation dispatch in IEEE14-bus.

Generator	Bus Type	Real Power [MW]	Reactive Power [Mvar]	Apparent Power [MVA]
G3	PV	120	-10	120.41
G6	PV	50	24	55.46
G8	PV	80	24	83.52
G-Slack	SL	19.16	-88.93	90.97
G2	PV	40	-40	56.56

Load flow and voltage stability studies were carried out using DIgSILENT [52] and VSAT [53]. The simulations for PV curves considered a set of N-1 contingencies for transformers and transmission lines, except transformers of generating units. For the CPF it is defined a generation and demand increase step. This step is distributed on every load of the system in proportion to its magnitude. N-1 contingencies are also performed.

Operating points of input matrix P for training is then obtained. VSAT generates files with voltage magnitude and angle for the base and contingency cases at each operating point along PV curves as in the second stage of Fig. 5. In the example, according to the demand step increase and number of points of PV curves for base case and contingencies, 2013 points were obtained. Extracting the points for testing the trained RBF, the number of columns, *a*, for the operational matrix P is defined. As will be explained, *a* will equal 2003 columns.

It has to be clear, that PV curves may have a different amount of points or columns according to the case, that is why the 2013 result is not a quantity obtained as a product of number of cases times number of PV points. For example, in the base case, 109 points were obtained to trace the PV curve. On the other hand, the number of rows varies according to the number of PMUs considered, for example, if 4 PMUs are located, P matrix will have 8 rows. P matrix would be 8 × 2003. In VSAT, when tracing the PV curves using an algorithm of a variable load step, in which fewer quantity of points are tackled along the less sharped parts of the PV curves, a reduction in the number of columns for P matrix is obtained. The reduction of P matrix accuses the concept of Dimensionality Reduction, DR, in Machine Learning, which is one of the requirements in model development.

Now, we will provide some comments according to the voltage stability studies for documenting the analysis.

It was found that bus number 14 is a weak bus as it exposed the lowest voltage support. For the N-1 contingencies simulated, the worst case was for the transformer 5–6 disconnection. In that case, the secure limit of the system was reached when the load demand exceeded 528 MW and it was the condition when voltage fell below 0.9 p.u. The voltage stability limit, which is a marginal point near collapse computed by VSAT (not exactly the theoretical nose point), was obtained when load demand exceeded 575 MW and voltage decreased below 0.78 p.u.

To obtain target matrix T, Voltage stability margins for each operating point and for the global network are calculated using (4) to (7). For this system, matrix T will be composed of secure and stability margins for each node. In this system, neither node 1 (slack), nor the 7^th^ (fictitious) are involved. Then, 12 nodes are considered for those margins, yielding 24 rows. Two additional rows are included for the global secure and stability margins. The total of rows for T matrix is then 26. T matrix has an order of 26 × 2003.

In order to evaluate the RBF estimation, judging the quantity of PMUs and specially the case that guarantees observability of the system, different cases are performed as a function of the number of PMUs, and verifying its performance in each training. An MSE of 0.7% is used for comparisons since it led to a training time of approximately 3 min for the base case of 13 PMUs. However, this criterion can be modified according to, e.g., SCADA system information actualization. Table 2 lists the results obtained when using PMUs in 13 nodes, in 4 arbitrary selected buses, in 3 nodes including the optimized location dictated by the observability theory, and finally the cases of 2 PMUs.

**Table 2:** RBF Sensitivities Results for IEEE 14 bus Test System. MSE = 0.7% and spread = 1.0.

Case	PMUs	Neurons	Training (s)	PMUs placement
1	13	451	167	All busbars
2	4	565	237	bus 2-6-9-14
3	4	585	245	bus 4-6-10-14
4	4	1498	2423	bus 6-9-11-13
5	3	1428	1742	bus 2-6-9
6	3	626	273	bus 2-6-14
7	3	-	Fail	bus 2-6-12
8	2	-	Fail	Several combinations

Table 2 shows that the cases with 2 PMUs for several combinations simulated did not lead to a convergence of an RBF. The conclusion is that margins could not be calculated using the data of two buses. It was found that the fastest case of margins calculation is, as waited, the one with PMUs in each bus, case 1. The four PMUs cases are different in training times. One of them is even slower than the full observability case with 3 PMUs, case 5. This shows that it is not a matter of PMUs quantity, but of network location. This conclusion is confirmed for a case of three PMUs not located according to observability method. This is the case number 6, in which location in buses 2, 6 and 14 needed much less training time than the placement based on observability.

To confirm that observability method is a start basis to define a reduced number of PMUs and to search for a suitable place for voltage stability monitoring, we show a case of three PMUs location that was not successful at ANN convergence, case 7.

It can also be found, that the number of neurons is correlated to the training time of the RBF.

Each designed RBF was trained considering 2003 operating points from the total 2013 simulated in VSAT. The remaining 10 points were used for validating the performance of the trained RBF. The criterion for the selection of the 10 operating points was that each point had to represent a different topological condition of the system. The selected points are in the positions: 5, 10, 150, 305, 710, 800, 900, 1405, 1900 and 2013. The total of topologies, families or PV curves were 19, included the base case and 18 contingencies. The 10 points used for validation, comprising voltage magnitude and angles in busbars with PMUs, were selected with the criterion that each point had to represent a different topological condition. These points reflect diverse qualities for the stability of the system, medium range of stability and close to the loss of stability where the system is mathematically complicated. For this, the absolute strategic ratio of training - validation of families could be termed as 10 topologies among 19.

**Fig. 8:**
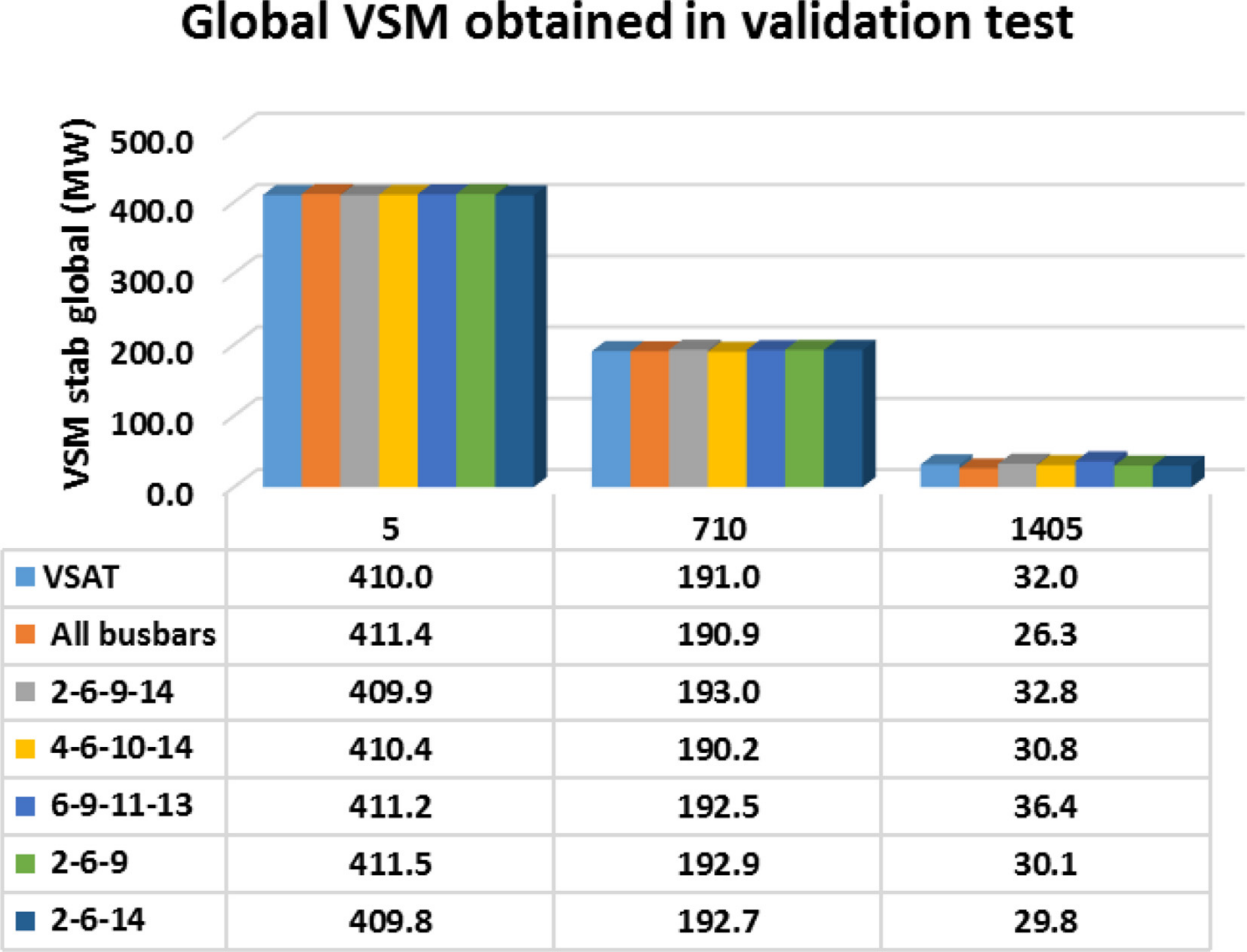
Global voltage stability margin estimation in the modified 14 bus IEEE system with the trained RBF for three operating points (5, 710 and 1405) and different PMU locations.

As an example, Fig. 8, shows the performance reached for each RBF in the estimation of global voltage stability margins for the points 5, 710 and 1405. Each pillar in Fig. 8 represents the estimated margin for the operating point tested, and the first pillar at each group represents the theoretical result (obtained with VSAT). From Fig. 8, it can be seen that the trained RBF meets with high accuracy the theoretical results.

The execution time was less than 1 s in an Intel ® Core ™ i7 3520 M CPU @2.9 GHz with 8 GB of RAM. This approach is promissory for real-time operation.

## 5 Results

The Antioquia area of Colombian system is composed of 61 buses in the 110 kV and 220 kV voltage levels. Antioquia is an area where most of generation resources are hydro. This is a main factor in the definition of two base cases for the stability studies. First, a high hydraulic generation scenario named HH includes a dispatch, where only 13% of resources are thermal. A low hydro generation scenario LH was set at 26% of thermal generation. Both base cases considered the same load demand in each bus. The optimal PMU placement in area Antioquia for 61 buses yields 15 buses with PMUs. Results are in Table 3.

**Table 3:** PMU optimal placement in antioquia area.

Bus	Voltage Level (kV)
Barbosa	220
Bello	110
Bolombolo	110
Chorodo	110
El Salto	110
El Tigre	110
Guatape	220
Guayabal	110
Itagui	110
Jaguas	220
La Clara	110
Occidente	110
Oriente	110
Villa Hermosa	110
Zamora	110

**Fig. 9:**
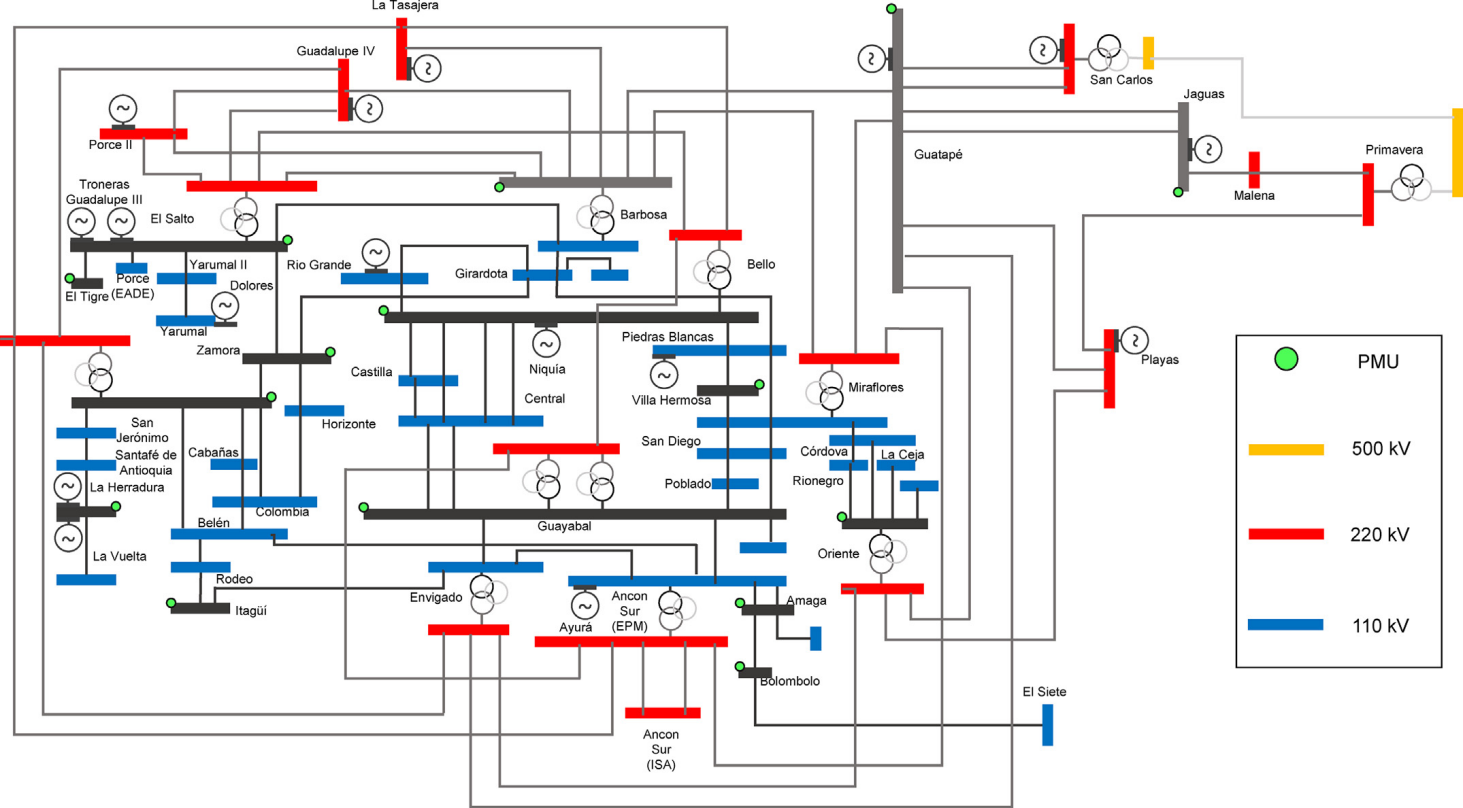
Antioquia area grid.

The Antioquia area is formed by transmission lines operating at 220 kV and 110 kV as shown in Fig. 9. It was necessary to proceed with a contingency ranking process and identify the lines with the highest impact in the voltage stability margin. VSAT includes a module that allows the execution of this process. For the analysis, this method was applied to determine the contingency ranking for HH and LH base cases.

For each of the base cases, the 10 most critical contingencies in transmission lines at 110 kV, and the 5 most critical contingencies in transmission lines at 220 kV were screened. The contingency set contained 32 elements including transmission lines and transformers. It means that voltage stability study is performed for each scenario in pre-contingency and considering each of the 32 contingencies.

The power transfer calculation was performed in VSAT applying load increases in Antioquia region, against a proportional increase in the local generators and in the rest of generators of the country. Antioquia load demand characteristic varies according to the hour of the day.

The combination of each load increase with pre and post-contingency cases for HH and LH scenarios generated a matrix of 6320 operating points. Additionally, for each operating point, the secure and voltage stability margins were calculated.

Input matrix used in ANN training is a 30 × 6220 arrangement where voltage magnitude and phase angle is tabulated for 15 substations in each of the 6220 operating points. The original matrix is a 30 × 6320 arrangement, but in order to test the final ANN, 100 operating points from input matrix are removed. Target matrix is a 96 × 6220 arrangement and contains secure and voltage stability margin for each load bus and for global area. The RBF was trained with Antioquia operating matrix points. The training was for MSE of 0.7%. The training took 1226.19 s, demanding 550 neurons.

**Fig. 10:**
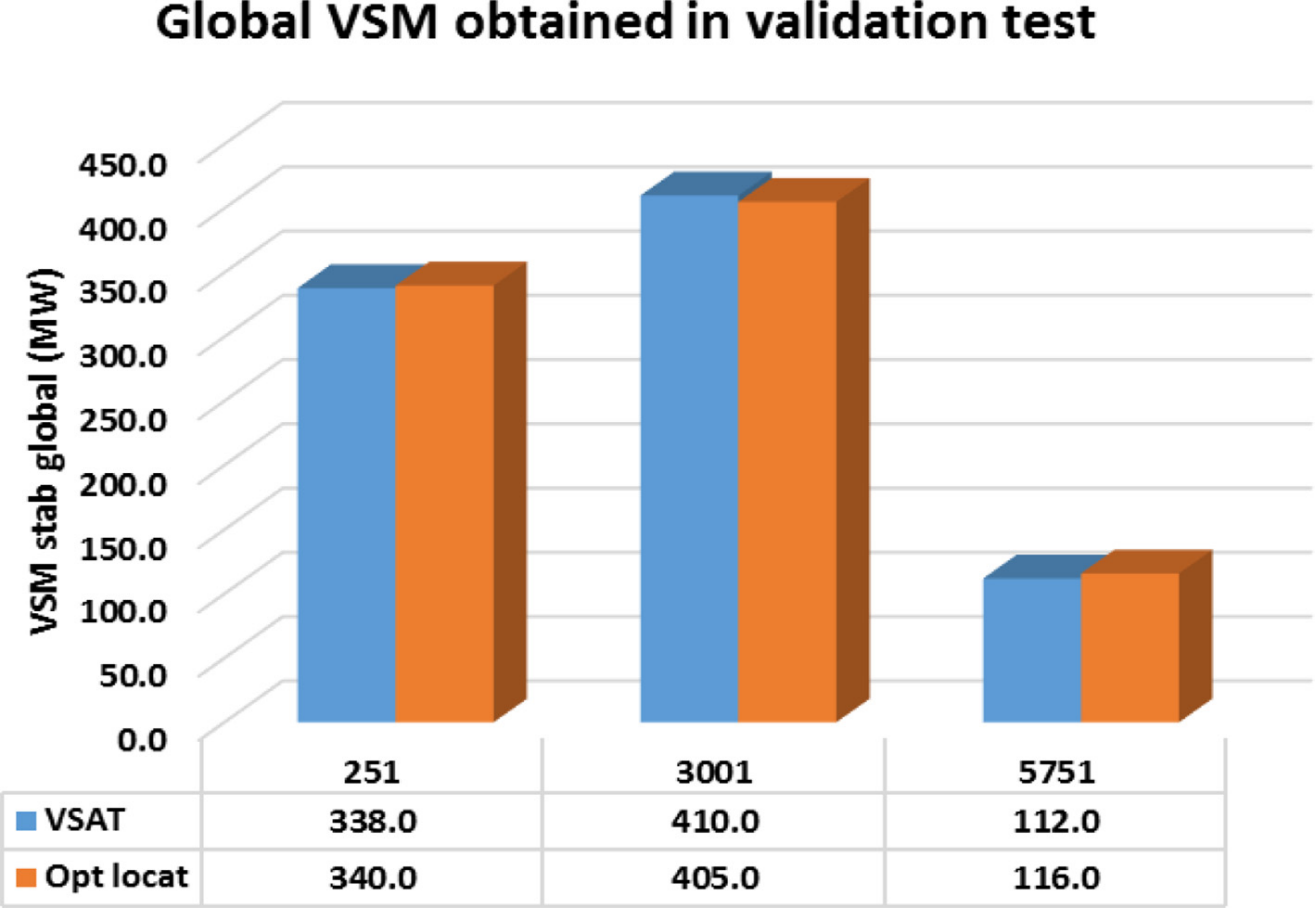
Global voltage stability margin estimation in the Antioquia area grid with the trained RBF for three operating points (251, 3001, 5751). VSAT and optimum location.

In the verification process, the 100 operating points that were not used in the training process, were used to evaluate the performance for each ANN. In Fig. 10 a bar chart illustrates the estimation of global voltage stability margin for each RBF trained. Only the 3 points, 251, 3001 and 5751, from the 100 operating points are displayed. The verification process carried out, took no more than 1 s using the same computer indicated in Section 4, that is why the method proposed is promissory for real-time implementation. From Fig. 10, the trained RBF meets with high accuracy the theoretical results.

## 6 Discussion and conclusions

A simple method based on Machine Learning Radial Basis Functions, RBF, was developed for estimating voltage stability margins in power systems. The method allows estimating active local and global power margins in normal operation and under contingencies. The proposed method is promissory for online applications due to its short execution time and since the trained RBF met with high accuracy the theoretical results.

The method utilized a reduced quantity of voltage phasors, including magnitude and also angle as input. Observability optimization technique for locating Phasor Measurement Units, PMUs, was applied.

To test the proposed method, validations in the IEEE 14-bus system and in a Colombian electrical network were done. In order to evaluate the RBF estimation, judging the quantity of PMUs and specially the case that guarantees observability of the system, different cases were performed as a function of the number of PMUs. The cases with less PMUs than the optimized quantity did not lead to convergence of RBF since margins could not be calculated using such little quantity of inputs. In general, when more phasors were available, the RBF training time was shorter. Nevertheless, there was a case in which the optimized number of phasors, having a lower quantity, was faster in training time that a case with one more phasor. This showed that the time in training is not only a matter of phasors quantity, but of PMU network location.

The latter conclusion was confirmed for a case in which a location not based on observability led to less training time than the case of placement based on the observability method. However, to reconfirm that the observability method is a good basis to define a reduced number of PMUs and a suitable place for voltage stability monitoring, we presented convergent and non-convergent cases using the same number of PMUs.

## Declarations

### Author contribution statement

Jorge W. Gonzalez, Idi A. Isaac, Gabriel J. Lopez, Hugo A. Cardona, Gabriel J. Salazar & John M. Rincon: Conceived and designed the experiments; Performed the experiments; Analyzed and interpreted the data; Contributed reagents, materials, analysis tools or data; Wrote the paper.

### Funding statement

This research did not receive any specific grant from funding agencies in the public, commercial, or not-for-profit sectors.

### Competing interest statement

The authors declare no conflict of interest.

### Additional information

No additional information is available for this paper.

## References

[1] Andersson G., Donalek P., Farmer R., Hatziargyriou N., Kamwa I., Kundur P., Martins N., Paserba J., Pourbeik P., Sanchez-Gasca J., Schulz R., Stankovic A., Taylor C., Vittal V. (Oct. 2005). Causes of the 2003 major grid blackouts in North America and Europe, and recommended means to improve system dynamic performance. IEEE Trans. Power Syst..

[2] Corsi S., Sabelli C. (2004). General blackout in Italy sunday September 28, 2003, h.03:28:00. IEEE/PES General Meeting, Denver.

[3] Kurita A., Sakurai T. (Dec. 1988). The power system failure on July 23, 1987 in Tokyo. Proc. 27th IEEE Conference on Decision and Control, Austin.

[4] Atputharajah A., Saha T. (Dec. 2009). Power system blackouts - literature review. International Conference Industrial and Information Systems (IICIIS), Sri Lanka.

[5] Beiraghi M., Ranjbar A. (March 2013). Online voltage security assessment based on wide-area measurements. IEEE Trans. Power Deliv..

[6] Lim J., DeMarco C. (July 2016). SVD-based voltage stability assessment from phasor measurement unit data. IEEE Trans. Power Syst..

[7] Corsi S., Taranto G. (Aug. 2008). A real-time voltage instability identification algorithm based on local phasor measurements. IEEE Trans. Power Syst..

[8] Smon I., Verbic G., Gubina F. (Aug. 2006). Local voltage-stability index using tellegen's Theorem. IEEE Trans. Power Syst..

[9] Torres S., Peralta W., Castro C. (Nov. 2007). Power system loading margin estimation using a neuro-fuzzy approach. IEEE Trans. Power Syst..

[10] Mansour M., Geraldi E., Alberto L., Ramos L. (Nov. 2013). A new and fast method for preventive control selection in voltage stability analysis. IEEE Trans. Power Syst..

[11] Parniani M., Vanouni M. (Feb. 2010). A fast local index for online estimation of closeness to loadability limit. IEEE Trans. Power Syst..

[12] Zarate L., Castro C., Ramos J., Ramos E. (Feb. 2006). Fast computation of voltage stability security margins using nonlinear programming techniques. IEEE Trans. Power Syst..

[13] Mansour M., Alberto L., Ramos R. (March 2016). Preventive control design for voltage stability considering multiple critical contingencies. IEEE Trans. Power Syst..

[14] Wang Y., Wang C., Lin F., Li W., Wang L., Zhao J. (Nov. 2013). Incorporating generator equivalent model into voltage stability analysis. IEEE Trans. Power Syst..

[15] Lee D. (Jan. 2016). Voltage stability assessment using equivalent nodal analysis. IEEE Trans. Power Syst..

[16] Xie L., Chen Y., Liao H. (Nov. 2012). Distributed online monitoring of quasi-static voltage collapse in multi-area power systems. IEEE Trans. Power Syst..

[17] Islam S., Sutanto D., Muttaqi K. (Sept. 2015). Coordinated decentralized emergency voltage and reactive power control to prevent long-term voltage instability in a power system. IEEE Trans. Power Syst..

[18] Liu X., Zhang X., Venkatasubramanian V. (March 2016). Distributed voltage security monitoring in large power systems using synchrophasors. IEEE Trans. Smart Grid.

[19] Li H., Bose A., Venkatasubramanian V. (March 2016). Wide-area voltage monitoring and optimization. IEEE Trans. Smart Grid.

[20] Bahmanyar A., Karami A. (June 2014). Power system voltage stability monitoring using artificial neural networks with a reduced set of inputs. Int. J. Electr. Power Energy Syst..

[21] Bahbah A., Girgis A. (May 2004). New method for generators' angles and angular velocities prediction for transient stability assessment of multimachine power systems using recurrent artificial neural network. IEEE Trans. Power Syst..

[22] Oludolapo O., Jimoh A., Kholopane P. (Aug. 2012). Comparing performance of MLP and RBF neural network models for predicting South Africa’s energy consumption. J. Energy South. Afr..

[23] Park J., Harley R., Venayagamoorthy G. (Aug. 2002). Comparison of MLP and RBF neural networks using deviation signals for on-line identification of a synchronous generator. Proc. IEEE Power Engineering Society Winter Meeting Conference, New York.

[24] Karami A., Mohammadi M. (2008). Radial basis function neural network for power system load flow. Int. J. Electr. Power Energy Syst..

[25] Moradzadeh B., Hosseinian S., Toosi M., Menhaj M. (July 2007). Online voltage stability monitoring and contingency ranking using RBF neural network. IEEE PES Power Africa 2007 Conference and Exposition, Johannesburg.

[26] Devaraj D., Roselyn J. (Nov. 2011). On-line voltage stability assessment using radial basis function network model with reduced input features. Int. J. Electr. Power Energy Syst..

[27] Lokman H., Moghavvemi M., Almurib H., Steinmayer O. (Oct. 2013). Current state of neural networks applications in power system monitoring and control. Int. J. Electr. Power Energy Syst..

[28] Jain T., Srivastava L., Singh S. (Nov. 2003). Fast voltage contingency screening using radial basis function neural network. IEEE Trans. Power Syst..

[29] Chakrabarti S., Jeyasurya B. (Jan. 2008). Multicontingency voltage stability monitoring of a power system using an adaptive radial basis function network. Int. J. Electr. Power Energy Syst..

[30] Baghaee H., Mirsalim M., Gharehpetan G., Talebi H. (Sept. 2018). Nonlinear load sharing and voltage compensation of microgrids based on harmonic power-flow calculations using radial basis function neural networks. IEEE Syst. J..

[31] Phadke A., Thorp J. (2008). Synchronized Phasor Measurements and Their Applications.

[32] Zima M., Larsson M., Korba P., Rehtanz C., Andersson G. (May 2005). Design aspects for wide-area monitoring and control systems. Proc. IEEE.

[33] Popovic D., Kukolj D., Kulic F. (July 1998). Monitoring and assessment of voltage stability margins using artificial neural networks with a reduced input set. IEE Proc. Gener. Transm. Distrib..

[34] Zhou D., Annakkage U., Rajapakse A. (Aug. 2010). Online monitoring of voltage stability margin using an artificial neural network. IEEE Trans. Power Syst..

[35] Liu M., Zhang B., Yao L., Han M., Sun H., Wu W. (2008). PMU based voltage stability analysis for transmission corridors. Third International Conference on Electric Utility Deregulation and Restructuring and Power Technologies, Nanjing, 2008.

[36] Jalboub M., Ihbal A., Rajamtani H., Abd-Alhameed R., Ihbal A. (2011). Determination of static voltage stability-margin of the power system prior to voltage collapse. Eighth International Multi-Conference on Systems, Signals & Devices.

[37] Ramirez L., Dobson I. (2015). Monitoring voltage collapse margin with synchrophasors across transmission corridors with multiple lines and multiple contingencies. IEEE/PES General Meeting, Denver.

[38] Han S., Lee B., Kim S., Moon Y. (2009). Development of voltage stability index using synchro-phasor based data. Transmission & Distribution Conference & Exposition: Asia and Pacific, Seoul, 2009.

[39] Han S., Lee B., Kim S., Moon Y., Chang B., Shin J. (2010). Voltage stability monitoring using PMU data in KEPCO system. IEEE PES T&D 2010, New Orleans.

[40] Kesherwani S., Singh S., Singh S. (2012). Voltage stability assessment using Phasor Measurement Units in power network with full system observability. 2nd International Conference on Power, Control and Embedded Systems, Allahabad, 2012.

[41] Gou B. (2008). Optimal placement of PMUs by integer linear programming. IEEE Trans. Power Syst..

[42] Gou B. (2008). Generalized integer linear programming formulation for optimal PMU placement. IEEE Trans. Power Syst..

[43] MathWorks (2017). Documentation Center, Matlab. https://www.mathworks.com/help/matlab/index.html.

[44] Cutsem T. (2000). Voltage instability: phenomena, countermeasures, and analysis methods. Proc. IEEE.

[45] Glavic M., Cutsem T. (2011). A short survey of methods for voltage instability detection. IEEE Power and Energy Society General Meeting, San Diego, 2011.

[46] Khatib A., Nuqui R., Ingram M., Phadke A. (2004). Real-time estimation of security from voltage collapse using synchronized phasor measurements. IEEE Power Engineering Society General Meeting, 2004, Denver.

[47] Milano F. (Aug. 2005). An open source power system Analysis Toolbox. IEEE Trans. Power Syst..

[48] Kundur P. (1994). Power System Stability and Control.

[49] Cutsem T., Vournas C. (1998). Voltage Stability of Electric Power Systems.

[50] Chen S., Cowan C., Grant P. (1991). Orthogonal least squares learning algorithm for radial basis function networks. IEEE Trans. Neural Netw..

[51] MathWorks (2017). Documentation Center, Neural Network Toolbox. http://www.mathworks.com/help/nnet/.

[52] DIgSILENT (2016). Power Factory User Manual Version 15.2.

[53] P. Labs (2013). DSA Tools-VSAT-Voltage Security Assessment Tool User Manual.

